# The Desert Duo: A Narrative Review on How Camel Milk and Date Fruits Combat Oxidative Stress and Enhance Metabolic Health

**DOI:** 10.3390/antiox15080945

**Published:** 2026-07-29

**Authors:** Hassan Barakat, Mohamed Ghonimy

**Affiliations:** 1 Department of Food Science and Human Nutrition, College of Agriculture and Food, Qassim University, Buraydah 51452, Saudi Arabia; 2 Department of Agricultural and Biosystems Engineering, College of Agriculture and Food, Qassim University, Buraydah 51452, Saudi Arabia

**Keywords:** camel milk, date fruits, oxidative stress, metabolic syndrome, antioxidant activity, glycemic control, functional foods, food supply

## Abstract

Metabolic syndrome is a multifactorial cardiometabolic disorder characterized by obesity, insulin resistance, dyslipidemia, hypertension, and oxidative stress. This narrative review evaluates the evidence for camel milk and date fruits as functional foods with potential antioxidant and metabolic benefits. A structured search of PubMed, Scopus, Web of Science, Google Scholar, and regional databases identified clinical, experimental, and review studies published from 2008 to 2026 on camel milk, date fruits, or their bioactive constituents in relation to oxidative stress and cardiometabolic outcomes. The available evidence suggests that camel milk may improve glycemic control, with reductions in HbA1c and insulin requirements reported in some trials. In contrast, date consumption generally does not impair glycemic regulation and may improve lipid and oxidative stress markers in selected settings. However, the evidence is heterogeneous with respect to study design, sample size, intervention form, dose, duration, cultivar, and participant characteristics. Preclinical findings support plausible antioxidant and anti-inflammatory mechanisms for both foods, but direct evidence for the combined intake of camel milk and dates is lacking. Overall, camel milk and date fruits may represent promising complementary dietary components, although standardized human trials are needed to confirm efficacy and clarify their interaction.

## 1. Introduction

Metabolic syndrome is a cluster of interrelated cardiometabolic abnormalities, typically including central obesity, hyperglycemia or impaired fasting glucose, hypertension, and atherogenic dyslipidemia, and it is used clinically to identify individuals at increased risk of type 2 diabetes and cardiovascular disease [1,2]. Its prevalence remains high worldwide, with pooled estimates varying by definition and region, and is particularly elevated in the Eastern Mediterranean, including the Middle East and North Africa, where rapid dietary and lifestyle transitions have contributed to an increasing burden of disease [3,4]. In Saudi Arabia and neighboring Gulf Cooperation Council countries, the shift toward energy-dense diets and reduced physical activity has further intensified the prevalence of metabolic syndrome and its components [5,6]. Because of its strong association with type 2 diabetes, atherosclerotic cardiovascular disease, and mortality, metabolic syndrome remains an important target for prevention and dietary intervention strategies [7,8].

Among the mechanisms implicated in metabolic syndrome, oxidative stress has received substantial attention because it can both reflect and amplify metabolic dysfunction [9,10]. Oxidative stress is characterized by an imbalance between reactive oxygen species (ROS) generation and antioxidant defenses, leading to oxidative damage to lipids, proteins, and nucleic acids and contributing to insulin resistance, endothelial dysfunction, and atherosclerosis [11,12]. Excess ROS can impair insulin signaling and promote inflammatory pathways, thereby worsening glycemic and lipid control and creating a vicious cycle with adipose dysfunction and vascular injury [13,14]. In this context, antioxidant enzymes such as superoxide dismutase and catalase, non-enzymatic antioxidants such as reduced glutathione (GSH), and oxidative damage markers such as malondialdehyde are commonly used to assess redox status in experimental and clinical studies [10,11,15].

Interest has therefore grown in food-based approaches that may help modulate oxidative stress and related metabolic disturbances [16]. Camel milk and date fruits (*Phoenix dactylifera*) are traditional foods in arid regions and have long been used in folk practice for nourishment, convalescence, and support of general health [17,18,19,20]. Camel milk contains whey proteins, bioactive peptides, vitamins, and minerals that may support antioxidant defense and glycemic regulation, while date fruits provide fiber, sugars, and phytochemicals with plausible redox and cardiometabolic relevance [17,18,21]. These foods are also culturally important in many communities, which makes them attractive candidates for regionally relevant nutritional strategies.

The rationale for considering camel milk and dates together is their potentially complementary nutritional profiles rather than established clinical interaction [22,23,24]. Camel milk provides protein, bioactive peptides, and minerals that may contribute to antioxidant and insulin-related pathways. In contrast, dates contribute fiber, phenolic compounds, and other phytochemicals that may influence postprandial metabolism, gut function, and lipid handling [22,25]. However, the available literature is fragmented: camel milk has been examined more often in human studies, particularly in relation to diabetes, whereas date research is broader but more heterogeneous, encompassing whole fruits, extracts, syrups, and date-containing products [21,26]. In addition, many experimental findings derive from *in vitro* or animal models, and their relevance to habitual human intake remains uncertain [18,26].

Accordingly, this review critically synthesizes the current evidence on camel milk and date fruits regarding oxidative stress and metabolic health, with emphasis on their nutritional composition, proposed mechanisms, human and experimental findings, and the limitations of the existing evidence base [16,21,26]. The review also aims to clarify what is supported by human data, what remains inferential, and where the most important research gaps lie, particularly regarding the absence of direct trials evaluating camel milk and dates together [21,26]. Therefore, this review addresses clinical and mechanistic data on the effects of camel milk and date fruits on metabolic health, including antioxidant markers, metabolic syndrome outcomes, potential synergies, and methodological gaps in the literature. It is designed to translate the evidence into culturally appropriate dietary guidance and future research directions and to determine whether this “desert duo” provides a viable adjunct to current approaches to metabolic disease.

## 2. Materials and Methods

This review was conducted as a narrative synthesis of the published literature on camel milk and date fruits in relation to oxidative stress and metabolic health. A structured search was performed in PubMed, Scopus, and Web of Science for studies published from January 2008 to June 2026, with earlier landmark papers added when needed to support background information on composition, traditional use, and mechanistic relevance. Search terms were developed around camel milk, date fruits (*P. dactylifera* L.), oxidative stress, antioxidants, metabolic syndrome, glycemic control, lipid profile, inflammation, and bioactive compounds, and were combined using Boolean operators.

A total of 447 records were identified across the three databases (PubMed: 142, Scopus: 210, and Web of Science: 95). After removing 110 duplicate records, 337 unique records remained for title and abstract screening, of which 270 were excluded because they were not directly relevant to camel milk or date fruits in relation to oxidative stress or cardiometabolic outcomes. Sixty-seven full-text articles were then assessed for eligibility, and the excluded studies were documented with reasons for exclusion, including lack of direct relevance to camel milk or date fruits in relation to oxidative stress or cardiometabolic outcomes, insufficient methodological detail, and a primary focus on non-nutritional uses.

Eligible records included randomized and non-randomized clinical trials, observational studies, systematic reviews, meta-analyses, and mechanistic or translational studies evaluating camel milk, date fruits, or their bioactive constituents in relation to antioxidant status or cardiometabolic outcomes. Studies were excluded if they were not relevant to the review question, lacked sufficient methodological detail, or focused on non-nutritional uses. Titles and abstracts were screened first, followed by full-text assessment of relevant records. From each included study, the following information was extracted where available: study design, population, sample size, intervention type, food form, dose, duration, comparator, and primary outcomes. Sources of heterogeneity, including camel breed, processing method, date cultivar, ripening stage, storage conditions, and background diet, were considered during interpretation. Because the included literature was heterogeneous in design, interventions, duration, and outcome measures, quantitative pooling was not appropriate; therefore, the evidence was synthesized qualitatively, with emphasis on nutritional composition, proposed mechanisms, human clinical findings, preclinical evidence, and the potential complementarity of camel milk and dates.

## 3. Nutritional and Bioactive Composition of Camel Milk and Dates

**Figure 1:**
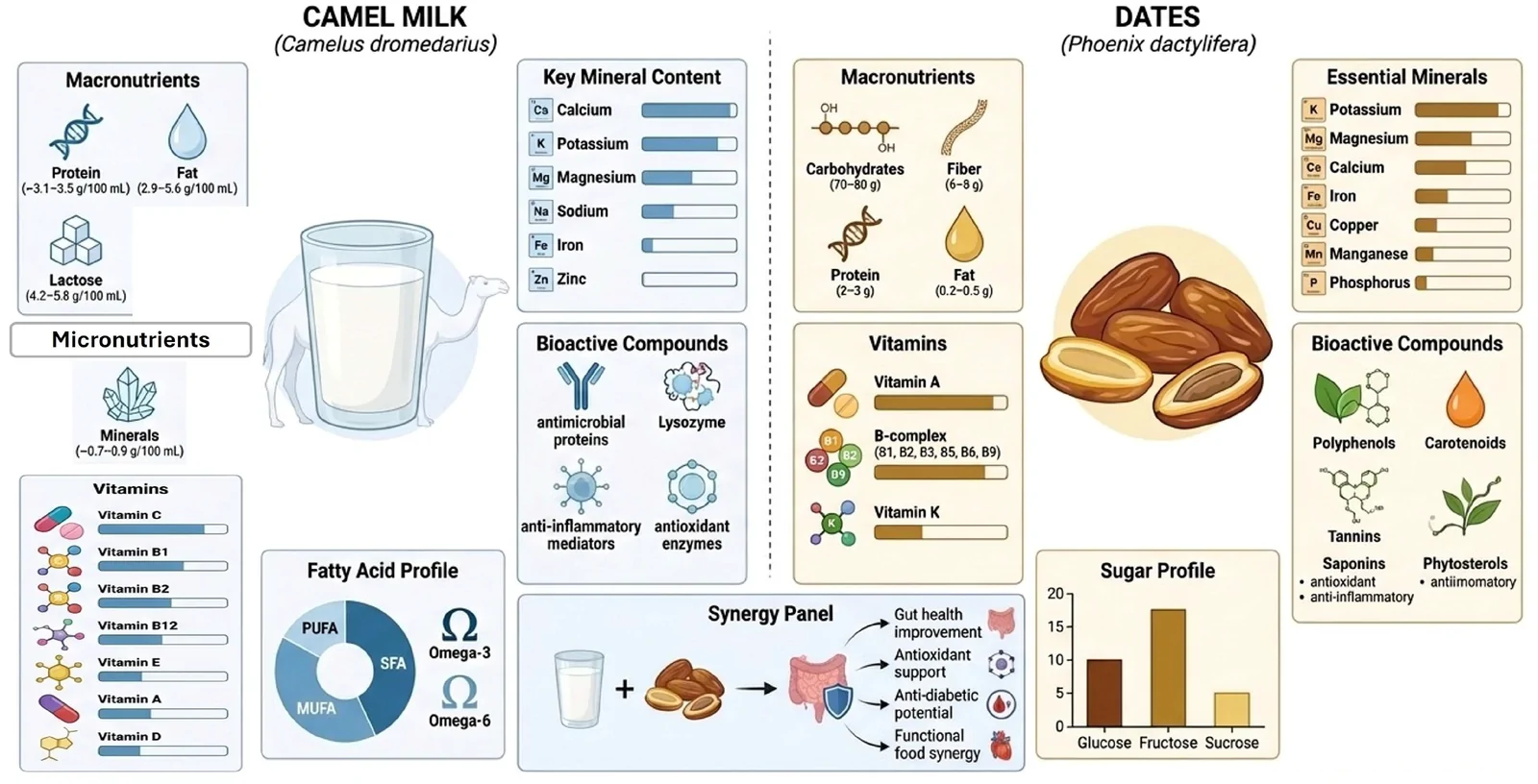
Conceptual framework illustrating how camel milk and date fruits may complement each other through macronutrients, bioactive components, and antioxidant-related mechanisms. The left panel summarizes the major macronutrients, vitamins, minerals, fatty acid profile, and bioactive compounds of camel milk (*Camelus dromedarius*), including immunoglobulins, lactoferrin, lysozyme, antimicrobial peptides, and insulin-like proteins. The right panel highlights the principal nutrients and phytochemicals in dates (*P. dactylifera*), including carbohydrates, fiber, vitamins, essential minerals, phenolics, carotenoids, tannins, saponins, and phytosterols. The central collaboration panel illustrates the complementary role of camel milk and dates in supporting gut health, antioxidant defense, antidiabetic potential, and functional food interaction.

Camel milk provides protein, fat, and a spectrum of bioactive proteins, including caseins, whey proteins, lactoferrin, immunoglobulins, lysozyme, lactoperoxidase, and peptide precursors with antioxidant, antimicrobial, antihypertensive, and antidiabetic activities [17,22,27,28]. Camel milk also supplies vitamin C and minerals such as zinc, magnesium, potassium, and iron, which support antioxidant defense systems and metabolic regulation [17,22]. During digestion or processing, these proteins can generate peptides with antioxidative, Angiotensin-converting enzyme (ACE)-inhibitory, and other functional activities, which may help explain why camel milk exerts effects beyond its basic nutrient composition [17,18,29,30]. However, compositional heterogeneity among raw, pasteurized, fermented, and powdered products complicates direct comparisons across studies and should be considered when interpreting reported health outcomes [31]. Dates are carbohydrate-rich fruits and sources of dietary fiber (mostly insoluble), phenolic acids (e.g., ferulic and caffeic), flavonoids, tannins, carotenoids, tocopherols, minerals, and B vitamins [32]. This phytochemical and fiber matrix provides a plausible basis for direct free-radical scavenging, modulation of postprandial glycemia, and broader cardiometabolic effects [25,26,33]. Date composition varies markedly with cultivar, agroecological conditions, ripening stage, storage, and processing, and differences in sugar profile, fiber content, and polyphenol concentration between soft, semi-dry, and dry forms are especially important when evaluating metabolic or antioxidant outcomes [32,34]. Consequently, any review of dates and health should distinguish generalized date consumption from effects attributable to specific cultivars or preparations [26]. As shown in Figure 1, camel milk and dates provide complementary nutrients and bioactive compounds that may support metabolic health through antioxidant, gut-related, and glycemic-regulating effects [35,36,37,38].

In addition, Table 1 compares the major nutritional and bioactive constituents of camel milk and date fruit to highlight their complementary roles in metabolic health. It helps the reader see at a glance how camel milk provides proteinaceous bioactives, key minerals, and insulin-supportive components.

## 4. Oxidative Stress and Metabolic Syndrome

Oxidative stress reflects an imbalance between oxidant generation and antioxidant defenses and is strongly implicated in the pathophysiology of obesity, insulin resistance, type 2 diabetes, dyslipidemia, endothelial dysfunction, and hypertension [10]. Reactive oxygen species can disrupt insulin signaling, promote inflammatory cascades, oxidize lipids and proteins, and impair vascular function, thereby contributing to the development and progression of metabolic syndrome [39]. Biomarkers commonly used to assess these processes include antioxidant enzymes such as SOD and CAT, non-enzymatic defenses such as GSH, oxidative damage markers such as MDA, and composite measures such as total antioxidant status or ferric reducing antioxidant power [40,41]. Dietary strategies that increase antioxidant capacity or reduce oxidative burden may therefore have metabolic relevance even when their clinical effects on weight, glycemia, or lipids are modest [42]. Camel milk and dates are both candidates for this role because they combine nutrient density with compounds that may act through overlapping but nonidentical pathways [17,18,43]. Camel milk emphasizes proteinaceous and micronutrient-mediated mechanisms, while dates emphasize fiber- and phytochemical-mediated mechanisms [27,44,45].

**Figure 2:**
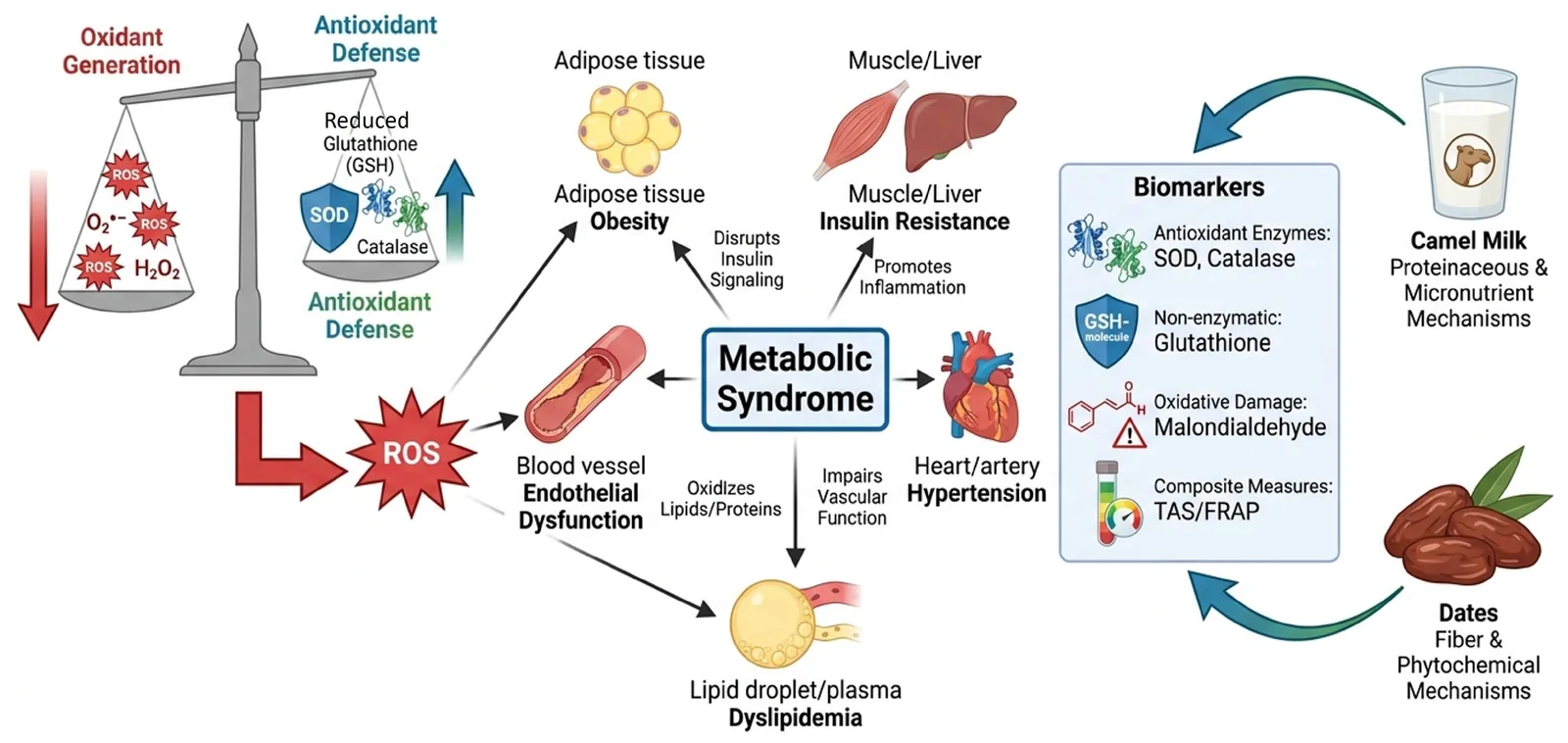
Role of oxidative stress in metabolic syndrome and dietary mitigation by camel milk and dates. The figure illustrates the imbalance between oxidant generation and antioxidant defenses, highlighting reactive oxygen species as a central driver of adipose tissue dysfunction, insulin resistance, dyslipidemia, endothelial dysfunction, and hypertension in metabolic syndrome. It also summarizes commonly used oxidative stress biomarkers, including antioxidant enzymes, GSH, MDA, and composite indices such as TAS/FRAP. Camel milk is depicted as a source of proteinaceous and micronutrient-mediated antioxidant support. In contrast, dates are shown to provide fiber- and phytochemical-mediated mechanisms that may help restore redox balance and mitigate metabolic dysfunction.

Oxidative stress is an important contributor to metabolic syndrome via an imbalance between oxidant production and antioxidant defenses, which may contribute to obesity, insulin resistance, dyslipidemia, endothelial dysfunction, and hypertension. The antioxidant activity of camel milk, including mechanisms involving proteins and micronutrients, may help alleviate these disturbances. In contrast, dates may support redox balance and metabolic regulation through their fiber and phytochemical content. Markers such as MDA, SOD, CAT, and glutathione peroxidase (GPx) are commonly used to characterize oxidative stress and antioxidant defense. In the context of camel milk and date fruits, changes in these biomarkers have been reported in animal and human studies, and Figure 2 summarizes the direction and magnitude of these effects.

## 5. Antioxidant and Metabolic Effects of Camel Milk

Camel milk has been widely discussed as a functional food with antioxidant, anti-inflammatory, hypoglycemic, and immunomodulatory properties [16,22]. Proposed mechanisms include the actions of lactoferrin and other protective proteins, the release of bioactive peptides, the provision of trace minerals needed for enzymatic antioxidant defenses, and the presence of insulin-like or insulin-supportive components. Experimental evidence also suggests that camel milk may attenuate inflammatory signaling and oxidative injury in animal models [18]. Human evidence is most developed in diabetes research, where randomized trials and meta-analyses suggest that camel milk can reduce glycated hemoglobin A1c (HbA1c) and insulin requirements and may lower fasting glucose in some settings [21,46]. A 2022 systematic review and meta-analysis of 14 randomized controlled trials involving 663 participants found significant reductions in HbA1c and insulin dose, while the reduction in fasting blood glucose showed a favorable but statistically nonsignificant overall trend [46]. More recent systematic review evidence also supports beneficial effects on glycemic indices, although heterogeneity across study design, duration, and product type remains substantial [46]. The lipid effects of camel milk seem to be more variable than glycemic outcomes [47]. Some of the trials show improvements in selected lipid parameters.

In contrast, pooled analyses indicate inconsistent changes in total cholesterol, triglycerides, LDL cholesterol, and HDL cholesterol, likely due to differences in baseline status, duration, dose, and comparator foods [21,47]. This pattern supports a cautious interpretation, in which camel milk may be an adjunctive aid for glycemic management. However, for cardiometabolic endpoints other than glucose control, stronger, more standardized evidence has yet to be collected [46,48].

**Figure 3:**
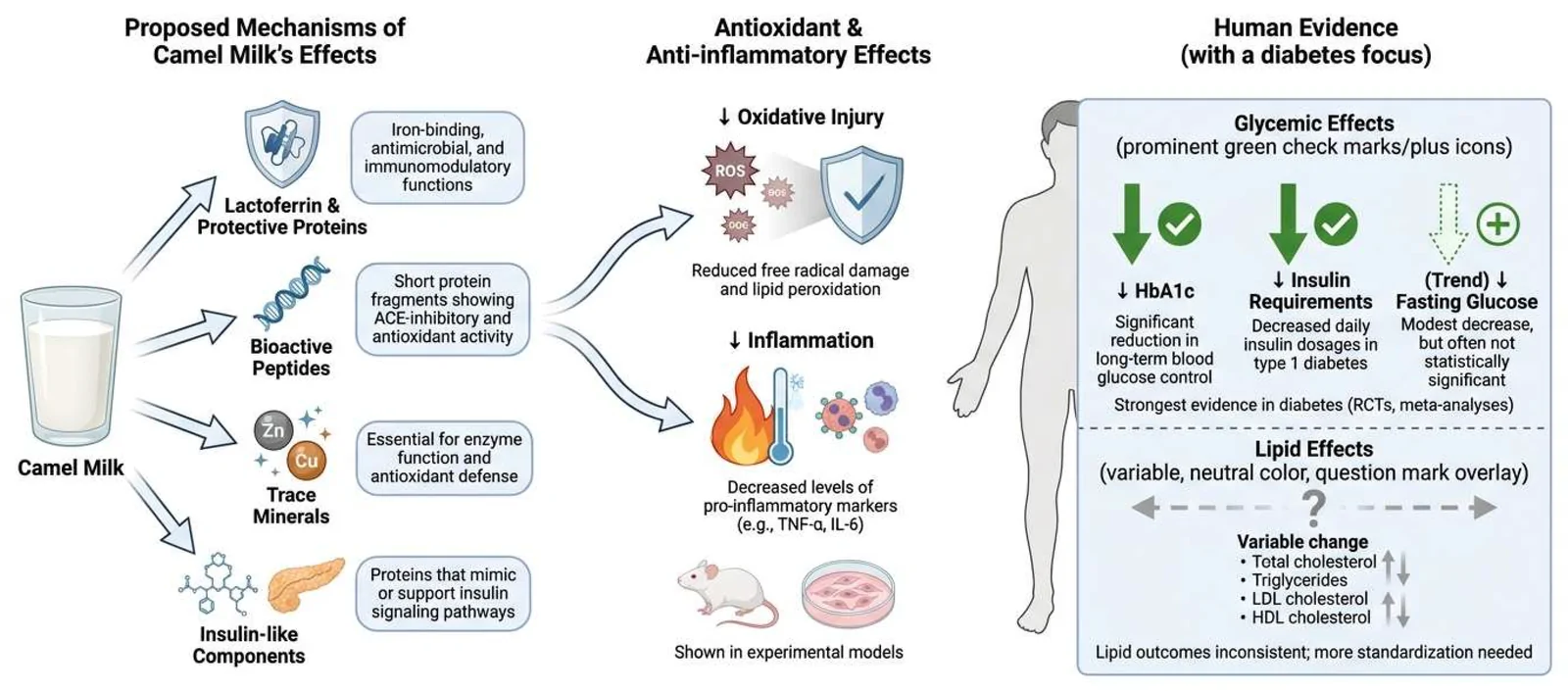
Camel’s milk—antioxidant and metabolic effects. The main proposed mechanisms of camel milk bioactivity: lactoferrin and other protective proteins, bioactive peptides, trace minerals, and insulin-like components. The figure also summarizes its antioxidant and anti-inflammatory actions, including reduced oxidative damage, lipid peroxidation, and inflammatory signaling in experimental models. The human evidence panel most consistently showed improvements in glycemic outcomes, such as HbA1c and insulin requirements, whereas the effects on lipids were variable and need further standardization and confirmation.

Camel milk has received attention for its potential roles in redox regulation, inflammation, and glycemic control. But the evidence is strongest for diabetes-related outcomes and less consistent for lipid effects. Figure 3 summarizes the proposed mechanisms, antioxidant and anti-inflammatory actions, and the main human findings, highlighting camel milk as a potentially useful adjunct food rather than a confirmed broad cardiometabolic therapy.

Table 2 summarizes the main human clinical studies and meta-analytic evidence on camel milk, showing that the strongest and most consistent benefits are reported for glycemic control, particularly HbA1c and insulin dose requirement. It also helps readers distinguish between more robust findings and outcomes that remain variable, such as fasting glucose, lipid parameters, and inflammatory markers, which is useful for interpreting the overall strength of the evidence.

## 6. Antioxidant and Metabolic Effects of Dates

Dates are often reported to be nutrient-dense fruits with high antioxidant potential, due to phenolics, flavonoids, tannins, carotenoids, and tocopherols [52]. The fiber content may also have metabolic effects by slowing carbohydrate absorption and supporting gut microbial fermentation, thereby improving dietary quality and satiety [53]. Mechanistically, dates may influence metabolic health by neutralizing reactive oxygen species, altering key inflammatory signaling pathways, and enhancing lipid and glucose metabolism [43,54,55].

**Figure 4:**
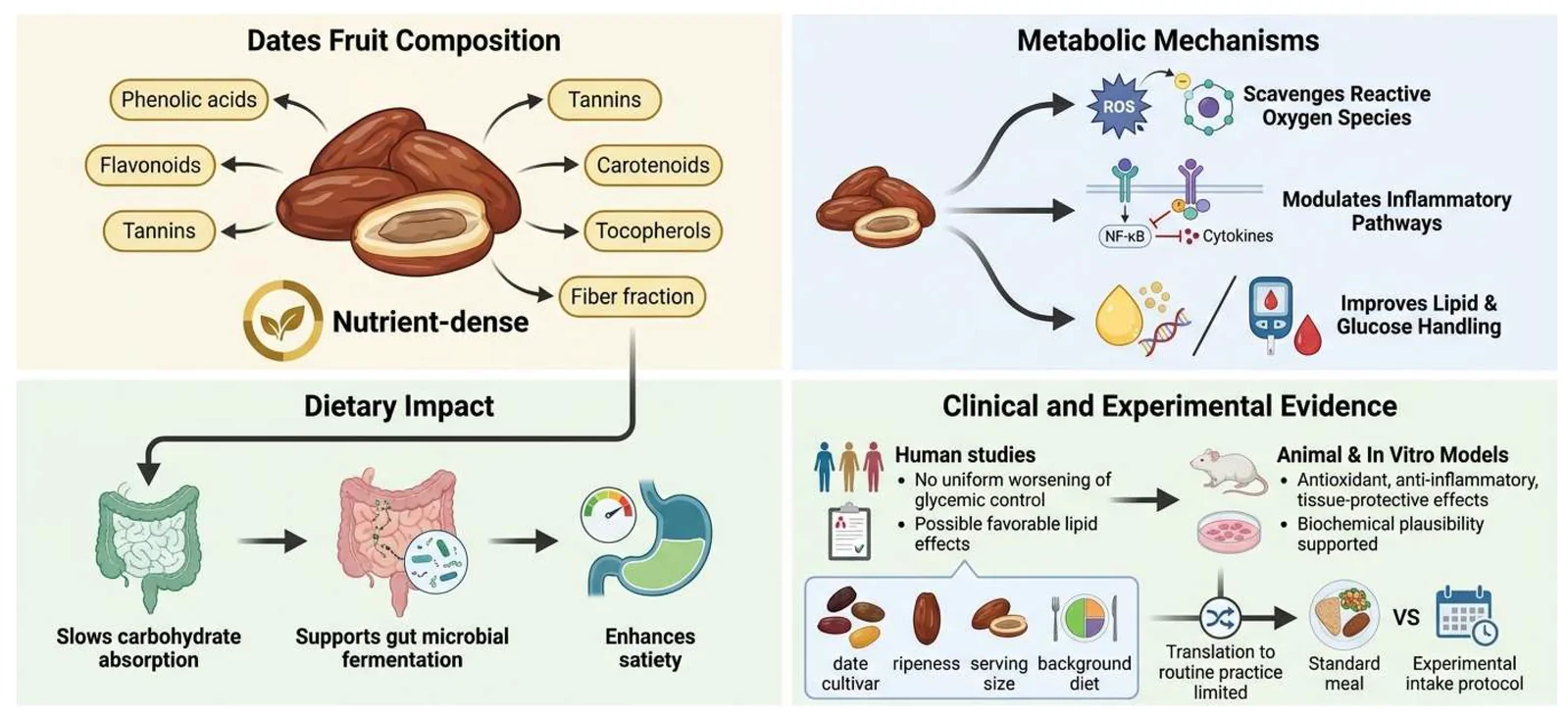
Dates, Antioxidants, and Metabolism. Diagrammatic representation of the proposed pathways by which date fruit consumption may influence metabolic health. Upper Left: Dates are represented as nutrient-dense fruits with high levels of phenolic acids, flavonoids, tannins, carotenoids, tocopherols, and a large fiber fraction. Upper right: These bioactives are believed to scavenge reactive oxygen species, modulate inflammatory signaling pathways (e.g., nuclear factor kappa B-regulated cytokines), and improve lipid and glucose handling. Lower Left: At the dietary level, the fiber and phytochemical matrix of dates may slow carbohydrate absorption, support gut microbial fermentation, and enhance satiety. Lower right: Summary of clinical and experimental evidence. Human studies do not generally demonstrate uniform worsening of glycaemic control and may suggest beneficial lipid effects. Animal and *in vitro* models support antioxidant, anti-inflammatory, and tissue-protective effects. The translation into routine practice is limited by factors such as date cultivar, ripeness, serving size, background diet, and intake protocol.

In 2024, a systematic review of date consumption and blood glucose and lipid outcomes in people with or without diabetes found that dates did not uniformly worsen glycemic control and may support favorable lipid effects in some contexts. However, the quality and consistency of studies were mixed [53]. Reviews also note that date cultivar, ripeness, serving size, and background diet are important modifiers of response, which limit broad generalization from findings from single studies [53,56]. Date fruit extracts or whole-fruit preparations have been frequently reported to possess antioxidant, anti-inflammatory, and tissue-protective effects in animal and *in vitro* studies [56]. These findings add to the biochemical plausibility of their use, but translation into routine human dietary practice remains limited, as many experimental models use concentrated extracts or conditions that do not reflect normal intake patterns [56]. Therefore, dates should be viewed as a potentially beneficial whole food rather than a consistently demonstrated therapeutic intervention [56]. However, to integrate these findings, we developed a conceptual model summarizing the potential influence of dates on metabolic health. The model links the characteristic phytochemical and fiber composition of date fruits with proposed antioxidant and anti-inflammatory mechanisms, downstream effects on glycaemic response and satiety, and the available clinical and experimental evidence on lipid and glucose homeostasis. This paradigm is based on dates as a nutrient-rich whole food with potential metabolic benefits and highlights the current limitations of human intervention data (Figure 4).

## 7. Complementary Bioactivity and Mechanistic Convergence

Camel milk and dates are plausible complementary foods when consumed together, as they provide different yet convergent nutrients and bioactive compounds [43]. Camel milk provides proteins, peptides, minerals, and protective proteins that may support antioxidant defenses, insulin action, and the control of inflammation. In contrast, dates supply energy, fiber, phenolics, and lipophilic antioxidants that may support redox balance, postprandial metabolism, and gut signaling [17,22]. This combination is especially relevant in traditional diets, where dates provide carbohydrates and camel milk supplies protein and micronutrients, improving the meal’s functional quality [57]. Mechanistic pathways suggest convergence. Glycemic effects may reflect date fiber and insulin-like components that influence glucose absorption, insulin requirements, and metabolic signaling [18,49]. Bioactive proteins and date phytochemicals may jointly reduce inflammation and oxidative stress linked to insulin resistance and vascular dysfunction [56]. The combination may influence lipid metabolism by altering the dietary matrix, hepatic pathways, and gut microbial activity.

**Figure 5:**
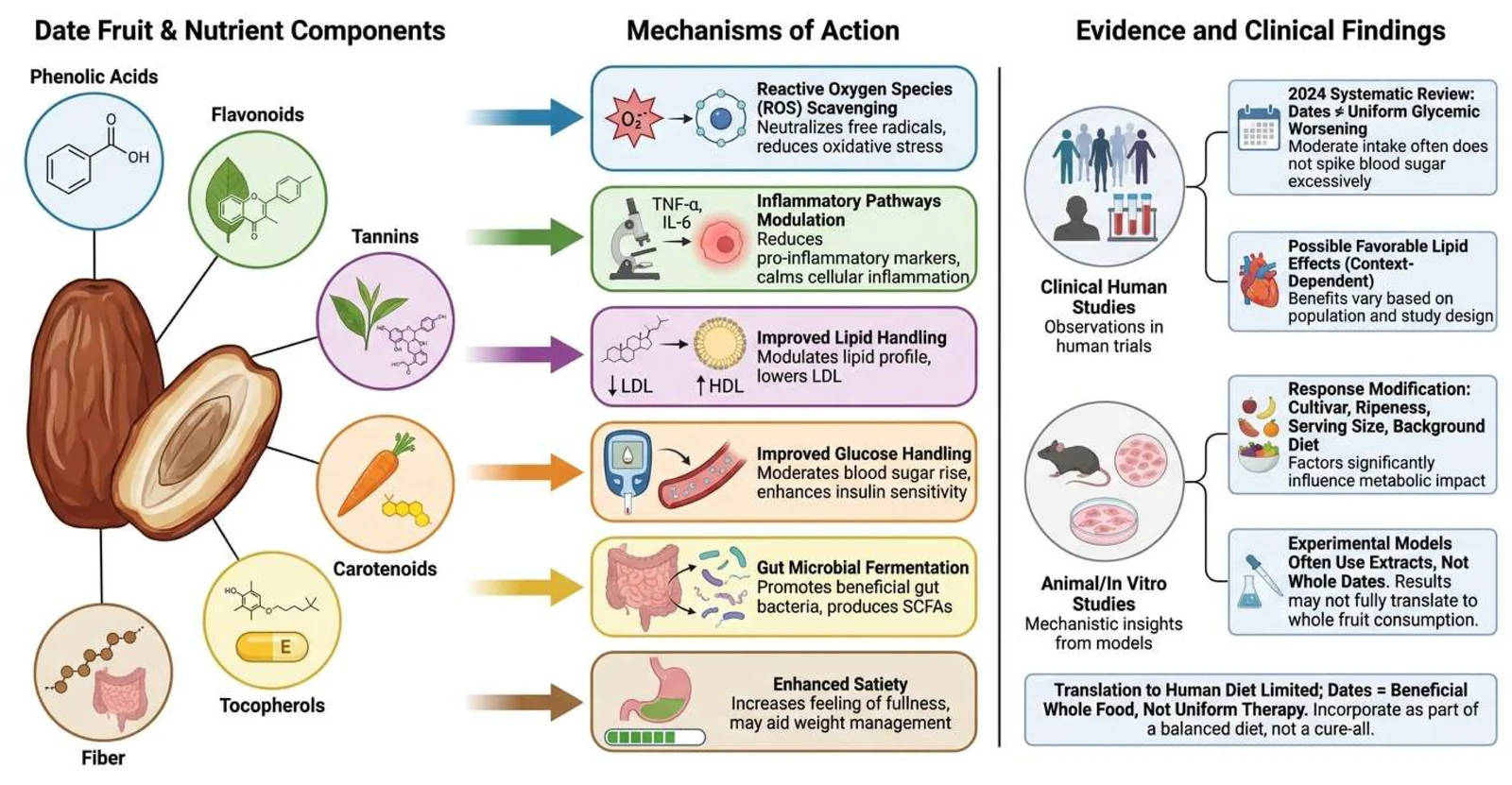
Date fruit—proposed antioxidant and metabolic effects. Dates are rich in phenolic acids, flavonoids, tannins, carotenoids, tocopherols, and dietary fiber. They may act through antioxidant, anti-inflammatory, glycemic, lipid, gut-related, and satiety-related pathways, while human evidence suggests that moderate intake is generally safe and may improve some lipid outcomes, although much of the mechanistic support remains preclinical.

Although no clinical trials have directly tested camel milk and dates together, their individual evidence suggests possible complementarity for antioxidant status, gut microbiota, and metabolic markers. This should be framed as a hypothesis-generating model, since any synergistic effect remains unproven and needs controlled intervention studies [58,59]. Accordingly, Figure 5 presents a conceptual model linking date composition with proposed metabolic effects and the current evidence base. It highlights potential roles for date bioactives, fiber, and lipophilic antioxidants in redox balance, inflammation, glucose and lipid homeostasis, gut fermentation, and satiety, while underscoring that human evidence remains context-dependent and insufficient for broad therapeutic claims.

## 8. Human and Animal Evidence

Human evidence is stronger for camel milk than for dates, especially for diabetes-related outcomes [46,60]. Randomized controlled studies suggest that camel milk may reduce fasting glucose and HbA1c, and in some trials, insulin requirements, although results remain heterogeneous and limited by small sample sizes, short duration, and variable protocols [46,49,50]. Dates have been studied in a wider range of forms, and available human data generally suggests that moderate intake does not worsen glycemic control and may improve lipid or antioxidant outcomes in some settings. Still, comparisons are difficult because of differences in cultivar, ripeness, dose, and study design [43,55,61,62,63]. Preclinical studies provide mechanistic support for both foods, showing antioxidant, anti-inflammatory, hypoglycemic, and microbiota-related effects. Still, these findings should be interpreted cautiously, as many are based on extracts or model systems rather than on normal dietary intake [43,44,59,61,64,65,66,67]. No human trial has directly tested camel milk and dates together, so any combined benefit remains hypothetical and should be evaluated in standardized randomized studies [57,62].

**Figure 6:**
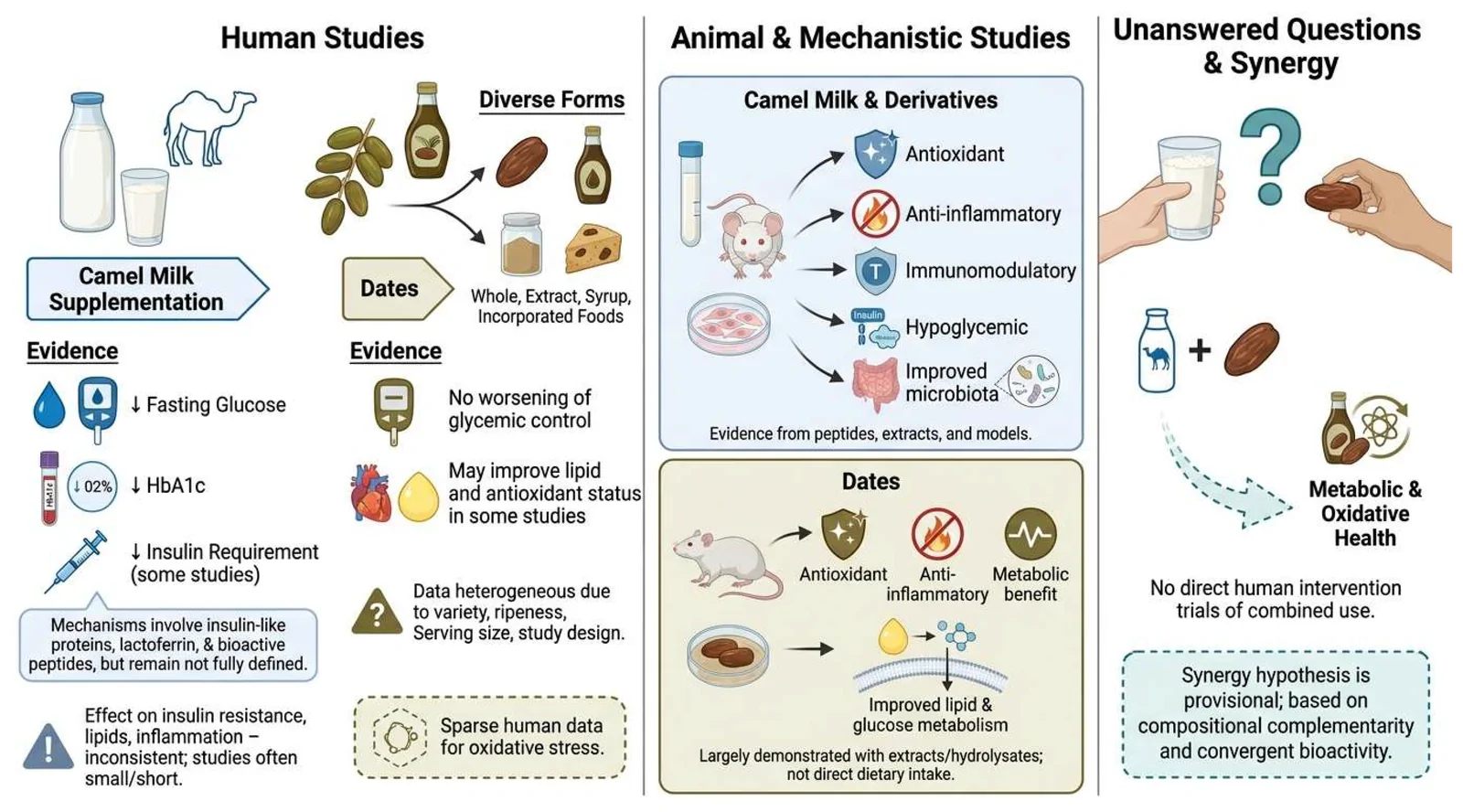
Camel milk shows the most consistent human evidence for improved glycemic outcomes, while dates appear safe and may provide complementary metabolic and antioxidant effects. Preclinical studies support antioxidant, anti-inflammatory, and microbiota-related mechanisms for both foods, but direct evidence for combined consumption is lacking; thus, the proposed combined effect remains unproven and requires standardized, randomized trials.

Camel milk may contribute to glycemic control and, in some cases, reduce insulin requirements in humans (Figure 6). Still, the results are inconsistent because the trials are small and differ in formulation and dose. Moderate consumption of dates appears safe and may slightly improve glucose, lipid, and oxidative stress parameters, and preclinical data suggest complementary bioactivities. However, no human studies have been performed to date to confirm the proposed interaction between camel milk and dates.

Table 3 summarizes the key human intervention studies and review-level evidence on date consumption. This overview facilitates the identification of reproducible, cultivar-dependent effects and informs appropriate portion sizes and study designs. It also helps disentangle the overall evidence base, distinguishing generally reassuring findings on glycemic control from more heterogeneous results regarding lipid profiles and oxidative stress biomarkers.

## 9. Limitations, Safety, and Research Gaps

The current evidence for camel milk and dates is promising, but interpretation remains constrained by substantial heterogeneity across studies. For camel milk, variability in product type, processing, dose, intervention duration, comparator, and participant characteristics limit cross-study comparability; for dates, differences in cultivar, ripeness, preparation form, serving size, and dietary context create similar challenges and may obscure true effects. Reporting quality is also inconsistent, with many studies failing to provide sufficient detail on biomarker selection, background diet, medication use, adherence, and co-interventions, thereby weakening the attribution of observed outcomes to the test foods and reducing reproducibility. Standardized reporting and more uniform study designs are therefore needed to strengthen interpretation.

From a safety perspective, camel milk is generally well-tolerated, but raw products raise concerns related to hygiene, microbial contamination, batch variability, and product standardization. Dates can be incorporated into a healthy diet as whole foods. Still, their relatively high carbohydrate content warrants caution in individuals with diabetes or marked insulin resistance, particularly when consumed in large amounts or in more concentrated forms such as juice or paste. Their glycemic impact depends not only on quantity but also on food form and the broader dietary context.

Important research gaps remain. Future work should prioritize adequately powered randomized controlled trials with clearly defined camel milk processing, specific date cultivars, standardized dosing, sufficient follow-up, and prespecified endpoints, including oxidative stress, inflammation, glycemic control, lipid profile, vascular health, and anthropometric outcomes. Direct combination studies are especially needed. At present, the proposed complementary effect of camel milk and dates remains biologically plausible but unproven, and comparative trials of camel milk alone, dates alone, and their combination are required to determine whether co-consumption produces additive or synergistic benefits.

## 10. Conclusions and Future Directions

This review indicates that camel milk and date fruits possess complementary bioactive profiles that could contribute to metabolic health by acting as antioxidant, anti-inflammatory, glycemic, and lipid-modulating agents. Human evidence is strongest for camel milk for glycemic improvement and for dates for metabolic safety and potential lipid/oxidative benefits. Preclinical data provide additional mechanistic support for both foods. These findings suggest that camel milk and dates may be culturally acceptable adjuncts to dietary management of metabolic syndrome, especially in populations that traditionally consume them. Interpretation, however, is limited by substantial heterogeneity in product type, processing, cultivar, dose, duration, and reporting quality, which hinders cross-study comparability and the development of firm clinical recommendations. Future studies should focus on adequately powered randomized controlled trials with standardized camel milk processing, precisely specified date cultivars, controlled doses, and extended follow-up durations. Outcomes should include oxidative stress, inflammation, glycemic management, lipid profile, vascular health, and anthropometric parameters, with direct comparisons between camel milk alone, dates alone, and the combination intervention arm. Such studies are necessary to determine whether the proposed camel milk–date interaction is additive, truly synergistic, or simply reflects overlapping nutritional benefits.

## Figures and Tables

**Table 1:** Selected nutritional and bioactive components of camel milk and date fruit and their functional relevance.

Component */Feature	Camel Milk (Typical per 100 mL)	Date Fruit (Typical per 100 g Edible Portion)	Functional/Metabolic Relevance
Energy	~60–70 kcal	~280–300 kcal	Supports energy balance; high-energy dates require portion control in metabolic syndrome
Protein	2.9–3.8 g, rich in whey proteins and caseins	~2–3 g, mainly albumins and globulins	Yield bioactive peptides with antioxidant, ACE-inhibitory, and hypoglycemic activities, while minor date proteins may still supply amino acids supporting antioxidant defenses.
Fat and fatty acids	~3–4 g; relatively higher proportion of long-chain unsaturated fatty acids than bovine milk	~0.2–0.5 g fat; small amounts of essential fatty acids	Milk lipids sustain cell membrane integrity and steroid hormone synthesis, whereas dates contribute only modest amounts of unsaturated fats and are not a significant fat source.
Carbohydrate	~4.5–5 g lactose	50–80 g total carbohydrate, mainly glucose, fructose, sucrose	Lactose provides a slower-release carbohydrate, while dates supply rapidly available sugars that modulate glycemic response.
Dietary fiber	Trace amounts	~7–11 g total dietary fiber (mostly insoluble) depending on cultivar and ripeness	Date fiber slows carbohydrate absorption, supports gut microbial fermentation, and may improve satiety and lipid handling.
Vitamin C	Often higher than bovine milk (e.g., ~30–40 mg/L)	Low to moderate levels	Camel milk vitamin C contributes to antioxidant capacity and may support redox balance.
Key minerals	Zn, Mg, K, Fe in physiologically meaningful amounts	K, Mg, Ca, Fe, and trace elements	Minerals act as cofactors for antioxidant enzymes (e.g., Zn for SOD, Fe for CAT) and support glucose and lipid metabolism.
Proteinaceous bioactives	Lactoferrin, immunoglobulins, lysozyme, lactoperoxidase, insulin-like proteins	Limited characterized protein bioactives	Exhibit antioxidant, anti-inflammatory, immunomodulatory, and insulin-mimetic effects in experimental models.
Phytochemicals	Modest levels of antioxidant peptides	Phenolic acids (e.g., ferulic, caffeic), flavonoids, tannins, carotenoids, tocopherols	Scavenges ROS, modulates inflammatory pathways, and may improve vascular and lipid markers.
Gut-modulating components	Peptides and proteins have been shown to affect gut microbiota in animal models	Fiber and SCFAs that act as substrates for beneficial microbes	Both foods can influence gut microbial composition and metabolites, potentially affecting glucose and lipid homeostasis.

*: Approximate values are expressed on a fresh-weight basis (camel milk per 100 mL; dates per 100 g edible portion) and may vary with camel breed, lactation stage, feed, processing method, date cultivar, ripening stage, storage conditions, and preparation form.

**Table 2:** Selected randomized and controlled clinical studies and meta-analyses of camel milk in diabetes and metabolic syndrome.

Study (Year)	Population (*n*, Condition)	Intervention (Camel Milk Form, Dose)	Comparator	Duration	Key Metabolic/Oxidative Outcomes
AlKurd et al. [46]	14 RCTs, 663 participants with type 1 and type 2 diabetes	Various camel milk preparations (fresh, pasteurized, fermented) were added to the usual care	Usual care without camel milk	4–24 weeks across trials	Pooled analysis showed significant reductions in HbA1c and insulin dose, with a nonsignificant trend toward lower fasting blood glucose
Sboui et al. [49]	60 adults with type 2 diabetes on oral hypoglycemic drugs	500 mL raw camel milk/day (two divided doses) plus usual medication	Usual medication only	3 months	The camel milk group showed reduced glucose, HbA1c, total cholesterol, and triglycerides, with no major renal safety concerns.
Fallah et al. [21]	40 adults with type 2 diabetes on long-acting insulin; 36 completed.	500 mL of raw camel milk/day alongside insulin therapy	500 mL of cow milk/day alongside insulin therapy	3 months	The camel milk group had lower fasting blood sugar and insulin dose, while changes in HbA1c and insulin resistance were less distinct between groups.
Agrawal et al. [50]	54 young patients with type 1 diabetes; 27 camel milk + usual care vs. 27 usual care	500 mL of camel milk/day added to standard insulin therapy	Standard insulin therapy alone	16 weeks	The camel milk group showed lower fasting blood sugar, HbA1c, insulin dose, anti-insulin antibodies, and urinary albumin, with higher C-peptide, suggesting improved insulin secretion.
Fallah et al. [51]	Adolescents with metabolic syndrome features	Fermented camel milk beverage	Control beverage or no intervention	Several weeks	Reported improvements in glycemic and inflammatory markers, but the sample was small, and endpoints were limited.

RCT, randomized controlled trial; HbA1c, glycated hemoglobin.

**Table 3:** Selected human trials and reviews on date consumption and cardiometabolic/oxidative outcomes.

Study (Year)	Population (*n*, Condition)	Date Product, Cultivar, and Dose	Duration	Main Outcomes Related to Glycemia, Lipids, and Oxidative Stress
Rock et al. [55], a pilot study in healthy adults	10 healthy subjects	100 g/day Medjool or Hallawi whole dates	4 weeks	Date intake did not increase fasting serum glucose or cholesterol; triglycerides decreased by 8–15% depending on variety, and oxidative stress markers (basal oxidative status and susceptibility to lipid peroxidation) improved, particularly with Hallawi dates.
Fallah et al. [21] RCT, low-dose dates in type 2 diabetes	100 adults with type 2 diabetes (pre- and T2DM; 39 men, 61 women)	3 dates/day (low-dose whole fruit) vs. avoidance of dates	16 weeks	The intervention group showed significant reductions in total cholesterol and favorable changes in HDL, with no worsening of HbA1c or BMI; quality-of-life scores improved, particularly mental health indices.
Al-Dashti et al. [52], date fruit and vascular health review	*In vitro*, animal, and limited human data on vascular endpoints	Whole dates and extracts, various cultivars, and doses	Not applicable	Synthesized evidence indicates that date fruit and extracts can modulate plasma lipids (triglycerides, cholesterol), oxidative stress markers, and inflammatory indices, with emerging human data suggesting potential vascular benefits but highlighting the need for rigorous clinical trials with standardized intake levels.
Awan et al. [43], a cardiovascular-focused systematic review of *P. dactylifera*	21 eligible studies, including *in vitro*, *in vivo*, and some clinical data	Whole dates and extracts across diverse preparations	Not applicable	Concluded that date fruit or extracts can beneficially influence lipid profiles, markers of inflammation, and oxidative stress, supporting their integration into cardiovascular-protective dietary patterns, while emphasizing gaps in dose–response characterization and long-term human outcomes.
Mirghani [63]	Patients with type 2 diabetes	Whole dates at culturally relevant portion sizes	Weeks to months	Generally, reports that moderate date consumption does not worsen glycemic control and may improve selected lipid parameters. However, study quality and reporting are variable and often limited by small sample sizes and heterogeneous protocols.

Study designs, date cultivars, serving sizes, and intervention durations vary widely across the included reports. RCT, randomized controlled trial; BMI, body mass index; HbA1c, glycated hemoglobin; HDL, high-density lipoprotein.

## Data Availability

No new data were created or analyzed in this study. Data sharing is not applicable to this article.

## Abbreviations

The following abbreviations are used in this manuscript:

Abbreviation	Meaning
ACE	Angiotensin-converting enzyme
BMI	Body mass index
CAT	Catalase
FBG	Fasting blood glucose
Fe	Iron
FRAP	Ferric reducing antioxidant power
GSH	Reduced glutathione
HbA1c	Glycated hemoglobin A1c
HDL	High-density lipoprotein
K	Potassium
LDL	Low-density lipoprotein
MDA	Malondialdehyde
Mg	Magnesium
PPG	Postprandial glucose
RCT	Randomized controlled trial
ROS	Reactive oxygen species
SOD	Superoxide dismutase
TAS	Total antioxidant status
TG	Triglycerides
T1DM	Type 1 diabetes mellitus
T2DM	Type 2 diabetes mellitus
Zn	Zinc
